# Antibodies against MERS Coronavirus in Dromedary Camels, United Arab Emirates, 2003 and 2013

**DOI:** 10.3201/eid2004.131746

**Published:** 2014-04

**Authors:** Benjamin Meyer, Marcel A. Müller, Victor M. Corman, Chantal B.E.M. Reusken, Daniel Ritz, Gert-Jan Godeke, Erik Lattwein, Stephan Kallies, Artem Siemens, Janko van Beek, Jan F. Drexler, Doreen Muth, Berend-Jan Bosch, Ulrich Wernery, Marion P.G. Koopmans, Renate Wernery, Christian Drosten

**Affiliations:** University of Bonn Medical Centre, Bonn, Germany (B. Meyer, M.A. Müller, V.M. Corman, D. Ritz, S. Kallies, A. Siemens, J.F. Drexler, D. Muth, C. Drosten);; National Institute for Public Health and the Environment, Bilthoven, the Netherlands. (C.B.E.M. Reusken, G.-J. Godeke, J. van Beek, M.P.G. Koopmans);; Erasmus Medical Centre, Rotterdam, the Netherlands (C.B.E.M. Reusken, J.F. Drexler, M.P.G. Koopmans);; EUROIMMUN AG, Lübeck, Germany (E. Lattwein);; Utrecht University, Utrecht, the Netherlands (B.-J. Bosch);; Central Veterinary Research Laboratory, Dubai, United Arab Emirates (U. Wernery, R. Wernery)

**Keywords:** Middle East respiratory syndrome, MERS, Middle East respiratory syndrome coronavirus, MERS-CoV, viruses, coronavirus, dromedary camels, camels, antibodies, serologic analysis, United Arab Emirates

## Abstract

Middle East respiratory syndrome coronavirus (MERS-CoV) has caused an ongoing outbreak of severe acute respiratory tract infection in humans in the Arabian Peninsula since 2012. Dromedary camels have been implicated as possible viral reservoirs. We used serologic assays to analyze 651 dromedary camel serum samples from the United Arab Emirates; 151 of 651 samples were obtained in 2003, well before onset of the current epidemic, and 500 serum samples were obtained in 2013. Recombinant spike protein–specific immunofluorescence and virus neutralization tests enabled clear discrimination between MERS-CoV and bovine CoV infections. Most (632/651, 97.1%) camels had antibodies against MERS-CoV. This result included all 151 serum samples obtained in 2003. Most (389/651, 59.8%) serum samples had MERS-CoV–neutralizing antibody titers >1,280. Dromedary camels from the United Arab Emirates were infected at high rates with MERS-CoV or a closely related, probably conspecific, virus long before the first human MERS cases.

## Abstract 2

Camels were infected with this virus >10 years before the first human cases.

Middle East respiratory syndrome coronavirus (MERS-CoV) is an emerging pathogen associated with severe respiratory symptoms and renal failure in infected patients (*1*,*2*). Globally, 156 laboratory-confirmed cases of infection with MERS-CoV, including 65 deaths, were reported as of early November 2013. All human cases were linked to the Arabian Peninsula (Saudi Arabia, Jordan, Oman, Qatar, Kuwait, and the United Arab Emirates). Imported cases were detected in countries in Europe and Africa (United Kingdom, Germany, Italy, France, and Tunisia) (*3*).

Transmission patterns, including the putative zoonotic source of the virus, remain unclear. Hypotheses include frequent zoonotic infections with limited subsequent human-to-human transmission chains and existence of a self-sustained epidemic in humans (*4*). A recent study found evidence to support the existence of epidemiologically unlinked cases in a large outbreak in the al-Hasa region, Saudi Arabia (*5*). It was speculated that zoonotic introductions of MERS-CoV from an unknown reservoir might occur at high rates, in addition to obvious human-to-human transmission.

Coronaviruses (CoV) are positive-sense RNA viruses. Viruses in the genera *Alphacoronavirus* and *Betacoronavirus* are associated with mammals and show a particularly high level of diversification in bats. Viruses in the genera *Gammacoronavirus* and *Deltacoronavirus* are mostly avian-associated viruses (*6*,*7*). MERS-CoV belongs to *Betacoronavirus* phylogenetic lineage C that, in addition to MERS-CoV, contains 2 distinct bat-associated CoV species (HKU4 and HKU5) (*1*,*8*).

Insectivorous bats of the family Vespertilionidae were recently shown to carry viruses that are probably conspecific with MERS-CoV (*9*). However, the limited rate of contact between humans and insectivorous bats makes a continuous and frequent acquisition of MERS-CoV from bats an unlikely scenario. In a manner similar to observations regarding severe acute respiratory syndrome CoV (SARS-CoV), an intermediate reservoir host might exist from which human infections are acquired. Dromedary camels from different regions in Africa and the Arabian Peninsula have been shown to have antibodies against MERS-CoV (*10*,*11*). Animals from the Arabian Peninsula had high neutralizing serum activities overall and reciprocal antibody titers ≤320–1,280, which support recent infection with MERS-CoV or a highly related virus. Thus, dromedary camels might serve as intermediate hosts. However, detailed serologic studies in countries with actual incidence of MERS-CoV infections in humans have not been conducted.

Serologic analysis of CoVs is challenging because of cross-reactivity between CoVs infecting the same host and the broad distribution of CoVs in diverse mammalian species (*6*,*7*,*12*–*14*). Antibodies directed against some of the major antigens of different CoVs are known to cross-react in standard serologic assays (*15*,*16*). Potential cross-reactivity is a diagnostic challenge because camelids are known to be infected with bovine CoV (BCoV), a distinct betacoronavirus of phylogenetic lineage A unrelated to the MERS-CoV (*17*,*18*). As an additional challenge, camel immunoglobulins lack a light chain peptide, which affects specific physical properties, such as altered size and stability, compared with immunoglobulins of other mammals (*19*,*20*). The influence of this feature on serologic assays has not been thoroughly investigated. Thus, serologic assays should be applied with caution, and different assay formats should be tested concurrently.

We reported a 2-staged approach for MERS-CoV serologic analysis in humans (*15*,*16*). Expanding upon these studies, we used in the present study a recombinant MERS-CoV spike protein immunofluroescence assay (rIFA) augmented by a validated protein microarray (*10*,*21*), followed by MERS-CoV–specific neutralization assay, to screen 651 dromedary camel serum samples from the United Arabian Emirates. Cross-reactivity against clade A betacoronaviruses was assessed by using a immunofluorescence assay (IFA) and a BCoV-specific neutralization assay. Serum samples obtained in 2003 and 2013 were compared to obtain information for the time in which MERS-related CoV has been circulating in camels.

## Methods

### Sampling

A total of 651 dromedary camel (*Camelus dromedarius*) serum samples were systematically sampled in Dubai, United Arab Emirates and the surrounding area in 2003 (collection 4, n = 151) and in 2013 (collections 1A, 1B, 2, and 3; n = 500). The total number of camels in that area was 360,000 in 2010 (*22*). Fecal samples were also available for collections 1A and 1B (n = 182), all obtained in 2013. Animals in collection 1B were born and raised at the Dubai Central Veterinary Research Laboratory, which tests ≈70,000 camels per year (*23*) and had no contact with other camels. Camels in collection 2 were racing camels (age range 2–8 years), and camels in collection 3 were adult livestock camels originally purchased from Saudi Arabia, Sudan, Pakistan and Oman.

Dromedary camel blood was obtained for routine health screening by jugular vein puncture according to standard veterinary procedures by trained personnel. For most serum samples, animal owners requested sample codes to be anonymous. All samples obtained during 2003 and 2013 were stored at −80°C until further analysis. For comparison, 16 serum samples from *C. bactrianus* camels in zoologic gardens in Germany were included in the study. All serum samples were shipped in agreement with German import regulations.

### Recombinant Spike IFA

For screening purposes, an rIFA was used (*15*,*24*). In brief, Vero B4 cells were transfected with pCG1 eukaryotic expression vector that contained the complete spike sequence of MERS-CoV or human CoV-OC43. Cells were fixed 24-h post-transfection with ice-cold acetone/methanol and stored dry at 4°C. Serum samples were applied at a dilution of 1:80 for 1 h at 37°C, which was optimal for reducing nonspecific reactions and maintaining sensitivity. Secondary detection was conducted by using a goat anti-llama IgG fluorescein isothiocyanate–conjugated antibody. For some negative serum samples, dilutions of 1:20 and 1:40 were also tested.

### Spike Protein Microarray

A confirmatory assay based on a protein microarray was performed as described (*10*,*21*) by using the spike S1 subunits of MERS-CoV, human CoV-OC43, and SARS-CoV. Serum samples were used at 1:20 dilutions on microarray chips. Relative light units were determined by using secondary cyanine 5–conjugated goat anti-llama IgG.

### MERS-CoV Conventional IFA

A MERS-CoV IFA with infected Vero cells was conducted as described (*15*) by using commercially available MERS-CoV IFA slides (EUROIMMUNAG, Lübeck Germany). Serum samples were used at dilutions of 1:20–1:5,120. Secondary detection was conducted by using goat anti-llama fluorescein isothiocyanate–labeled IgG (1:200 dilution; Agrisera, Vännas, Sweden).

### Serum Neutralization Test

Serum neutralization tests were conducted as described (*10*) by using Vero B4 (MERS-CoV) or PT (BCoV) cells. To reduce volumes of serum needed, all neutralization tests were performed in a 96-well format. Reactions contained 50 PFUs of MERS-CoV (EMC/2012 strain) or BCoV (Nebraska strain) in 25 μL of medium mixed 1:1 with camel serum diluted in 25 μL serum-free Dulbecco minimum essential medium. The starting dilution was 1:40. After incubation for 1 h at 37°C, each well was infected for 1 h at 37°C with a 50 μL virus–serum mixture. Supernatants were removed and fresh complete Dulbecco minimum essential medium was added. Assays were terminated by fixation with 8% paraformaldehyde for 30 min and stained with crystal violet after 3 days. Neutralization titers were defined as serum dilutions reducing cytopathic effects in 2 parallel wells.

### Detection of Virus Nucleic Acid

Viral RNA was extracted from serum and fecal samples by using the MagNA Pure System (Roche, Basel, Switzerland) and an input volume of 100 μL of serum or fecal material suspended 1:10 in phosphate-buffered saline buffer. The elution volume was 100 μL for serum and fecal suspensions. To identify CoV-specific nucleic acids, 2 generic CoV PCRs were performed as described (*25*–*27*), followed by subsequent Sanger sequencing of the amplified DNA.

## Results

To characterize reactivity of camel serum samples with MERS-CoV in different assay formats, we chose 11 camel serum samples with weak and strong reactivity predetermined by using a simple IFA. The 11 serum samples were titrated in a 2-fold dilution series in all applied assays. The reactivity pattern of the MERS-CoV spike protein (MERS-S) was compared against that of the human CoV-OC43 spike protein (OC43-S). As in our previous study (*10*), human CoV-OC43 was used instead of BCoV in these initial experiments because it is serologically indistinguishable from BCoV and is not subject to handling restrictions of German Animal Diseases Protection Act (*28*). Overall titers against MERS-S were higher than those against OC43-S, and several serum samples reacted exclusively against 1 of the 2 viruses (Table 1), suggesting the absence of general cross-reactivity between spike proteins of both viruses by IFA. Typical patterns of reactivity observed for camel serum samples are shown in the Figure, panel A.

**Table 1:** Validation of serologic assays for coronaviruses with differentially reactive dromedary serum samples, United Arab Emirates, 2013*

Serum no.	rIFA titer†‡			Protein array (RFU) ‡§				vIFA titer†‡		Neutralization test titer¶#	
	MERS-S	OC43-S		MERS-S1	OC43-S1	SARS-S1		MERS-CoV		MERS-CoV	BCoV
1	–	–		2,555	3,868	2,606		–		–	40
2	–	320		2,770	18,896	2,776		–		–	80
3	–	640		3,950	65,535	2,751		–		–	160
4	320	–		65,535	3,921	1,726		640		40	–
5	>10,240	320		65,535	7,247	2,306		>5,120		2,560	160
6	5,120	640		65,535	5,069	2,098		2,560		640	160
7	>10,240	160		65,535	7,179	2,198		>5,120		640	40
8	5,120	320		65,535	55,826	2,412		>5,120		1,280	160
9	5,120	>5,120		65,535	65,535	2,087		>5,120		1,280	320
10	>10,240	320		65,535	22,695	2,303		>5,120		1,280	320
11	5,120	1,280		65,535	28,391	2,858		>5,120		640	40

*rIFA, recombinant immunofluorescence assay (antigen used was complete spike protein); RFU, relative fluorescence units; vIFA, Middle East respiratory syndrome coronavirus–based immunofluorescence assay (antigen used complete virus); MERS-S, spike protein from Middle East respiratory syndrome coronavirus; OC43-S, spike protein from human coronavirus OC34; SARS-S, spike protein from severe acute respiratory syndrome virus; MERS-CoV, Middle East respiratory syndrome coronavirus; BCoV, bovine coronavirus; –, negative.  †Serum dilutions started at 1:20.  ‡Assay was used for screening purposes. §RFU <4,000 were considered negative. The serum dilution used in this assay was 1:20 (antigen used was S1 subunit of spike protein). ¶Serum dilutions started at 1:40. #Assay applied for confirmation purposes.

**Figure:**
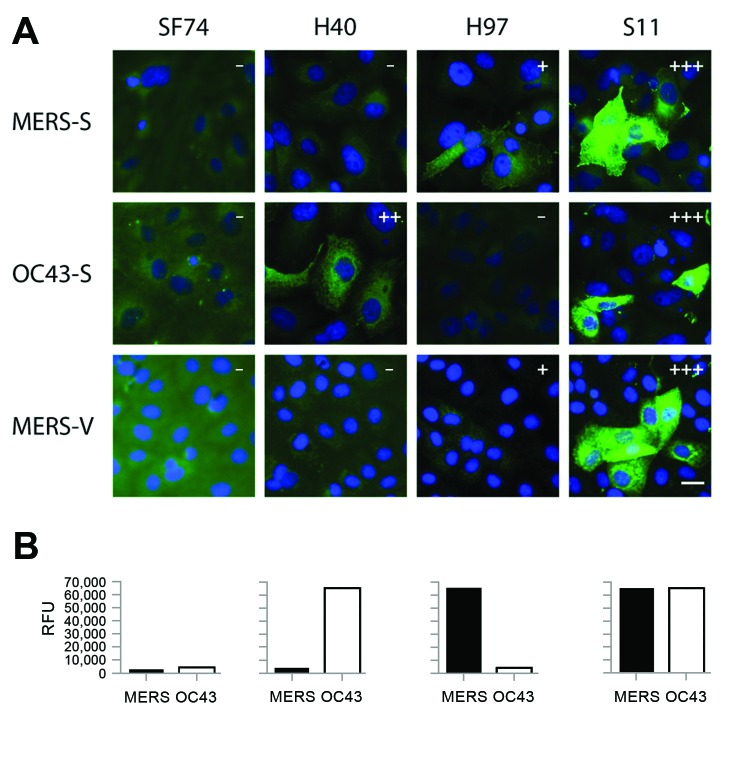
Immunofluorescence and microarray reactivity patterns for antibodies (SF74, H40, H97, and S11) against Middle East respiratory syndrome coronavirus (MERS-CoV) in serum samples from camels, United Arab Emirates, 2013. A) Serum samples tested against overexpressed MERS-CoV spike protein (MERS-S), overexpressed human CoV-OC43 spike protein (OC43-S), and Vero cells infected with MERS-CoV (MERS-V). Fluorescence intensities were evaluated as follows: –, negative; +, weakly reactive; ++, reactive; +++, strongly reactive. Scale bar indicates 20 μm. B) Relative fluorescence units (RFU) were determined for the same serum samples by microarray using S1 domains of MERS-CoV and human CoV-OC43.

A previously published microarray-based assay that used the receptor-binding S1 spike subunit of MERS-CoV (MERS-S1), human CoV-OC43 (OC43-S1), and SARS-CoV (SARS-S1) was also evaluated. In contrast to our previous studies (*10*,*21*) we chose a lower fluorescence intensity cutoff of 4,000 instead of 20,000 relative fluorescence units (RFU) to maximize the sensitivity and thereby challenge the target specificity. All 3 MERS IFA-negative serum samples had signal intensities <4000 RFU at serum dilutions of 1:20 (Table 1). All rIFA-positive serum samples had saturated signals >65,535 RFU. The OC43-S1 reactivity pattern across the serum panel was comparable with that for the OC43-S rIFA. As expected, all serum samples were negative against the SARS-S1 control antigen. A comparison of typical reactivity patterns in the microarray with those of the IFA is shown in the Figure, panel B. Results for the rIFA and protein microarray were highly congruent.

The panel of camel serum samples was additionally tested in a commercially available IFA that used cells infected with MERS-CoV (vIFA) (EUROIMMUN AG). The use of whole virus provides additional structural and nonstructural protein antigens, including envelope, membrane, nucleocapsid, and diverse replicase proteins. However, because of conserved features of nonstructural proteins among even distantly related CoVs (*7*,*12*), cross-reactivity was possible with this assay (*15*). In the tested panel of camel serum samples, vIFA titers corresponded well to titers determined by rIFA and generally equal to or higher than titers in the rIFA (Table 1). Despite the absence of cross-reactivity between MERS-S–positive and OC43-S–positive serum samples in this test (Figure, panel A), in previous studies the vIFA showed false-positive results with human CoV-OC43–positive serum samples, in particular if used at lower dilutions, such as 1:10 or 1:20 (*15*,*16*).

To confirm results from affinity assays with results from a functional test, we determined endpoint virus neutralization titers by using a microneutralization test against MERS-CoV and BCoV. In most animals MERS-CoV serum neutralization titers were higher than titers against BCoV (serum samples 4–11) (Table 1). High IFA titers generally corresponded with high neutralization titers, with exceptions for some BCoV antibody–positive serum samples. Divergence between affinity and neutralization assays can result from waning neutralizing antibody activity for infections that occurred long ago. Neutralization assays confirmed the absence of cross-neutralization between MERS-CoV and BCoV antibodies in either direction even at low dilutions, such as 1:40. However, sample no. 1 (Table 1) neutralized BCoV at a dilution of 1:40 despite showing negative results in all other serologic assays. This finding indicates that nonspecific neutralization activities might be encountered with camel serum samples, suggesting that higher serum dilutions should be used when conducting critical investigations such as viral reservoir studies.

On the basis of the validation studies, we investigated 4 collections of serum samples from dromedary camels from the United Arab Emirates that were sampled in 2003 and 2013. For initial screening, we chose the rIFA because of its proven sensitivity and decreased chances of generating false-positive results. All 667 camel serum samples from the United Arab Emirates and Germany were initially screened at dilutions of 1:80. A total of 89.0%–100.0% of serum samples in 4 collections showed positive results (Table 2). Seroprevalence was higher for collections from exclusively adult animals (collections 3 and 4) than for a collection from young racing camels (2–8 years of age, collection 2). Clear seropositive results included 151 dromedary camel serum samples obtained in 2003 (collection 4). All 16 serum samples from German zoologic gardens were tested at the same dilution and showed no reactivity in the rIFA. Re-testing at lower dilutions of 1:20 and 1:40 confirmed absence of reactivity in these serum samples. Subcollection 1B contained serum samples from 5 animals that were born in, and had never left, a closed animal research facility in Dubai; these animals were seronegative.

**Table 2:** MERS-CoV serologic results for dromedary serum and fecal samples, United Arab Emirates, 2003 and 2013*

Collection	Year	No. camels/ sex	Camel age	Feature	No. samples	Serum dilution, no. (%) positive				
						rIFA, MERS-S†		Neutralization test, MERS-CoV		
						80		<640	640–1,280	>1,280
1A	2013	2/M, F	A, J	Paired serum and fecal samples	177	175 (98.9)		24 (13.6)	74 (41.8)	79 (44.6)
1B	2013	2/M, F	A, J	Animals raised at CVRL	5	0		5 (100.0)	0	0
2	2013	2/M, F	2–8 y	Racing camels	100	89 (89.0)		55 (55.0)	3 (3.0)	42 (42.0)
3	2013	2/M, F	A	Livestock camels‡	218	217 (99.5)		23 (10.6)	13 (6.0)	182 (83.5)
4	2003	1/F	A	Systematically sampled	151	151 (100.0)		35 (23.2)	30 (19.9)	86 (57.0)
Total					651	632 (97.1)		142 (21.8)	120 (18.4)	389 (59.8)

*MERS-CoV, Middle East respiratory syndrome coronavirus; rIFA, recombinant immunofluorescence assay with MERS-CoV spike protein; MERS-S, spike protein from MERS-CoV; A, adult; J, juvenile; CVRL, Dubai Central Veterinary Research Laboratory. †Fluorescence signal intensity was rated as negative, +, ++, +++, and ++++. ‡Originally purchased from Saudi Arabia, Sudan, Pakistan, and Oman.

A confirmatory microneutralization test was conducted at dilutions of 1:640 and 1:1,280 for all IFA-reactive serum samples. These high dilutions were chosen on the basis of our observation of high levels of neutralizing serum activity in camels (*10*). Most (59.8%, 389/651) serum samples had high neutralizing titers >1,280 (Table 2). In 18.4% (120/651) of all serum samples, neutralization titers ranged from 640 through 1,280, and 21.8% (142/651) of rIFA-positive serum samples had neutralizing titers <640.

To rule out cross-reactivity and to study additional exposure of MERS-CoV–positive camels with BCoV (*17*,*18*), all serum samples having MERS-CoV neutralizing titers >640 were tested by using a BCoV-specific microneutralization assay. At a dilution of 1:640, a total of 19.2% (23/120) of MERS-CoV–neutralizing serum samples had concomitant neutralizing activities against BCoV (Table 3). Of serum samples that had MERS-CoV neutralizing antibody titers >1,280, a total of 24.2% (94/389) had concomitant neutralizing activities against BCoV.

**Table 3:** BCoV neutralization test results for MERS-CoV–positive dromedary serum samples, United Arab Emirates, 2003 and 2013*

Collection	No. BCoV positive/no. MERS-CoV positive (serum dilution, %)	
	640–1,280	>1,280
1A	15/74 (20.3)	14/79 (17.7)
1B	0	0
2	0/3 (0.0)	14/42 (33.3)
3	2/13 (15.4)	52/182 (28.6)
4	6/30 (20.0)	14/86 (16.3)
Total	23/120 (19.2)	94/389 (24.2)

*BCoV, bovine coronavirus; MERS-CoV, Middle East respiratory syndrome coronavirus.

Fecal samples were available for 182 dromedary camels in collection 1. All samples were tested by using a subfamily *Coronavirinae*–specific broad-range reverse transcription PCR (RT-PCR) and a highly sensitive RT-PCR specific for genus *Betacoronavirus* phylogenetic lineage C. Both assays were specific for the viral RNA-dependent RNA polymerase gene. Two positive fecal samples were identified by both assays. Sequencing of amplified cDNA fragments of 182 nt and 404 nt identified sequences 99% identical with BCoV strain Mebus (GenBank accession nos. KF894801 and U00735.2). To further confirm virus identity, we amplified a region within the spike protein gene (positions 24303–24702 in BCoV strain Mebus) by using RT-PCR and sequencing it. Amplicons from both animals were 97% identical at nucleotide level with BCoV strain Mebus, indicating the presence of BCoV in camels as reported (*10*). We tested all serum samples in the same way by RT-PCR and obtained uniformly negative results.

## Discussion

We have shown that dromedary camels from the United Arab Emirates, a country with human cases of MERS-CoV infection, have antibodies that can neutralize MERS-CoV at high rates. Antibodies were detected in serum samples obtained in 2013 and in serum samples obtained >10 years earlier, which indicated the long-standing presence of MERS-CoV or a closely related virus in dromedary camels in that region. Our data add to previous studies in which our group and others have reported wide antibody prevalence in camels in various regions, including Oman, Egypt, and the Canary Islands (*10*,*11*). A 10% lower seroprevalence in collection 2, which contained young racing camels, suggests that animals might be infected as juveniles. However, because only limited data were made available by owners, a definite statement awaits confirmation.

The absence of antibodies in a control cohort from Germany might be explained by the fact that these animals belonged to a different camelid species (*C. bactrianus* vs. *C. dromedarius*). However, because MERS-CoV has a highly conserved receptor structure, we did not assign high priority to the hypothesis that the closely related camel species *C. bactrianus*, should be less susceptible than *C. dromedarius* camels to MERS-CoV (*29*,*30*). Differences in antibody prevalence rates might reflect a restricted geographic distribution of the virus, which corresponds to our previous finding of a relatively lower prevalence of antibodies against MERS CoV in camels from the Canary Islands, which have been isolated from their point of origin in Africa for many years (*10*). Therefore, MERS-CoV–like viruses in camelids might be spreading across a region covering at least the eastern Arabian Peninsula, including Oman, the United Arab Emirates, Egypt, and Morocco from where some of the antibody-positive camels described by Reusken et al. originated (*10*).

The high rates of antibody prevalence in contemporary serum samples and samples from 2003 suggest that the virus has spread in camelids for some time. However, recognition of camelids as the bona fide reservoir for MERS-CoV has to await sequencing of camelid-associated MERS-related CoV. In this context, only animals infected with conspecific viruses can be regarded as reservoirs for a given virus. Although neutralization assays can provide evidence of infection with a virus belonging to the same serotype, no systematic studies have defined whether serotypes correlate with CoVs species. Nevertheless, for several CoV clades, serotypes defined by neutralization assay will not include >1 viral species. Members of the species *Betacoronavirus 1*, including CoV-OC43 and BCoV, show cross-neutralization with each other, but the closely related sister species (human CoV-HKU1) does not show cross-neutralization (*31*).

Feline CoV (FCoV) comprises 2 subserotypes that show limited cross-reactivity but are considered 1 virus species. Transmissible gastroenteritis virus of swine shows more efficient cross-neutralization with 1 of these FCoV subserotypes than the other and is classified as 1 species with FCoV even though it is carried by a different host (*32*). Human CoVs 229E and NL63, which form 2 closely related sister taxa, do not show cross-neutralization and concordantly form 2 different species by genetic criteria (*33*). Therefore, our finding of high neutralizing antibody titers in camelids is suggestive (but not evidentiary) of the presence of viruses conspecific with MERS-CoV in camelids. Final confirmation will depend on the identification of virus sequences in camelids, which should expectably be closely related to human-specific MERS-CoV sequences.

Camels probably acquired MERS-CoV at some unknown time. Potential sources include bats of the family Vespertilionidae, in which a virus with a close phylogenetic relationship with MERS-CoV has been detected (*9*). This virus, which is carried by vespertilionid bats of the genus *Neoromicia*, has been confirmed to be conspecific with MERS-CoV. Lineage C betacoronaviruses in other bat taxa have also been proposed to be related to MERS-CoV (*34*,*35*). However, although these viruses cluster phylogenetically with MERS-CoV, they are not conspecific with MERS-CoV on the basis of sequence distance criteria, such as that were proposed by Drexler et al. (*36*).

In vespertilionid bats, including those in the genus *Neoromicia*, virus conspecific with MERS-CoV differs from human MERS-CoV, even if formally a member of the same species. The observed degree of sequence divergence between this virus and MERS-CoV makes any direct and recent transmission from bats to humans seem unlikely. Nevertheless, it cannot be excluded from available data that the virus source population in bats has not been detected. For example, a recent investigation of *Rhinolophus* bats in China identified viruses with close relationships to the bona fide ancestor of SARS-CoV, and viruses described in many studies yielded only conspecific yet less related viruses (*37*). In that study, viruses from civet cats, which are deemed to be intermediary hosts in the transition of SARS-CoV from bats to humans, were still more closely related to human SARS-CoV than even the closest bat-borne virus.

If camelids should function as intermediary hosts in a similar manner, we should expect a virus in camelids that has a closer phylogenetic relationship with any bat-borne CoV and thus should be easily detectable with available RT-PCRs. Larger studies to confirm the presence of MERS-CoV in camelids should receive high priority so as to define the animal reservoir of MERS-CoV and possibly control it by such measures as vaccination or control of animal movement. However, before implementation of any control measures, whether camelids are a continuous source of infection for humans needs to be firmly established.
